# Narrowing down dimensions of e-learning readiness in continuing vocational education — perspectives from the adult learner

**DOI:** 10.3389/fpsyg.2022.1033524

**Published:** 2022-10-28

**Authors:** Vanessa Stefanie Loock, Jens Fleischer, Anne Scheunemann, Linda Froese, Katharina Teich, Joachim Wirth

**Affiliations:** ^1^Research Group of Research on Learning and Instruction, Ruhr-University Bochum, Bochum, Germany; ^2^Research Group of Psychology of Education, Ruhr-University Bochum, Bochum, Germany; ^3^Research Group of Educational Psychology and Technology, Ruhr-University Bochum, Bochum, Germany

**Keywords:** e-learning, adult learners, SRL, e-learning readiness, vocational education

## Abstract

Although e-learning has become an important feature to promote learning experience, still little is known about the readiness of adult learners for e-learning in continuing vocational education. By exploring perceived challenges and benefits, it was our aim to identify dimensions that define e-learning readiness. Therefore, we conducted a study design with qualitative and quantitative components. It consisted of both, semi-structured interviews, as well as an online survey regarding biography, personality, learning behavior, and general attitudes toward e-learning. The continuing vocational education course that we were investigating comes from the field of project management. The learner group was heterogeneous regarding their biographical and occupational background. Our results suggest several dimensions of e-learning readiness which are namely: motivation, learning strategies/regulation, attitudes toward learning, and personality-associated aspects as well as digital literacy. These findings are in line with previous research to only some extent, but reveal the necessity to redefine single dimensions of e-learning readiness to develop an inventory that is generalizable for different adult learner groups. Based on these assumptions a new measure for e-learning readiness needs to be proposed in future research as a next step.

## Introduction

E-Learning (electronic learning) is nowadays widely established in different kinds of learning environments. Also in adult continuing and vocational education, there was a massive increase in the use of e-learning during the COVID-19 pandemic worldwide (Stracke et al., 2022). Many educational courses had to be transferred into a digital format within a short time period to make learning possible even in phases of lockdown. It can be assumed that also post-pandemic more educational institutions will stick to e-learning (Abi-Raad and Odhabi, 2021).

This rapid transition from in-person attendance learning environments to e-learning settings is best described for schools and applied to many countries worldwide (Al-Nuaimi and Al-Kabi, 2021). Within the group of 10 to 15-year old learners there was a sevenfold growth in using e-learning over the last two years in Germany, for example (Statistisches Bundesamt, 2020). It is still under debate whether and how this forced increase of e-learning impacts the learners with regard to their individual e-learning readiness levels. That applies all the more to adult learners in continuing vocational education, as probably not all were used to e-learning before (Kulikowski et al., 2021). It is questionable whether or not adult learners are able to adapt to these new requirements in the same pace and without previous instruction or training. As recent research about this specific group of learners is still scarce, it is our aim to contribute to a better understanding of the adult learners’ needs by identifying e-learning readiness dimensions.

### E-learning

As there are different synonyms for e-learning such as online learning, digital learning, distance learning, or computer-based learning (see also Moore et al., 2011), it first needs clarification what is meant by e-learning exactly. A widely accepted and general definition of e-learning was suggested by Kerres (2013, p. 6). He described e-learning as “generic term for all variants of the use of digital media for teaching and learning purposes.” Different types of e-learning have evolved over time. For example, there are synchronous and asynchronous settings or a combination of both. While in a synchronous setting all learners work on the same topic at the same time, in asynchronous learning settings learners are free to choose when they work on a topic. Often only a deadline is given (Shahabadi and Uplane, 2015).

Also hybrid course formats are available in continuing vocational education. This allows higher levels of flexibility regarding time and place to learn and meets the requirements of adult learners better, who often have social and more intense work obligations. One common hybrid teaching method in e-learning is the flipped classroom model, where the learners start their course with a digital self-study phase, before they meet in (digital) class (see Uzunboylu and Karagozlu, 2015 for a literature review).

E-learning environments impose specific requirements on the learner such as having access to necessary and useful technology, being able to create an appropriate learning environment, and having the ability and willingness to learn more autonomously (Fotiadou et al., 2017). Many adult continuing education courses are open to a wide range of participants, so that such kind of learner groups may be very heterogeneous. With the growing shift toward e-learning within the last two decades, more research has been conducted and brought diverse groups of learners and problems arising from that fact to attention. Anderson (1997) noted that in non e-learning technical and vocational education, student groups vary in their amounts of work experience, motivation, language skills and numeracy levels and concludes that different learning needs exist. This should even more apply to e-learning contexts. Also, the cultural background of learners may influence participation with peers (Mittelmeier et al., 2018).

The general effectiveness of e-learning has been researched extensively. Means et al. (2013), for example, conducted a meta-analysis finding that e-learning is even more effective compared to classroom settings. Pei and Wu (2019) found no differences between online and offline learning settings regarding learning effectiveness in higher education. By a systematic review, Müller and Wulf (2020) identified numerous antecedents that determine learning effectiveness, namely learner, teacher, format, and technology characteristics. The findings are inconsistent with regard to different outcome variables. However, there is evidence, that even when confronted with identical learning conditions such as curricula or learning setting, the learners’ individual learning success in terms of learning achievement and course satisfaction may differ (e.g., Castillo-Merino and Serradell-Lopez, 2014; Eom and Ashill, 2016; Baber, 2020). Both, achievement and satisfaction, are important for further educational decisions of the learner and can influence each other. Ye et al. (2022) could prove a positive relationship of course satisfaction with self-assessed learning effectiveness. Booker and Rebman (2005) found that satisfaction is also related to achievement improvement. Levy (2007) and Rajabalee and Santally (2021) postulate that satisfaction is important for successful learning and decreases the chance of course drop-out. However, we emphasize that especially course satisfaction is relevant for assessing learning success in adult continuing education as often no objective and valid measures of learning achievement are available. Therefore, we will focus on bringing dimensions of e-learning readiness into relationship with course satisfaction as outcome criterion in the present study.

### E-learning readiness

We suggest that the reasons for the previously mentioned differences in learning outcome can be traced back to a learners’ individual level of e-learning readiness among other variables. Only learners who are ready or prepared for e-learning will succeed in their individual learning process (Guglielmino and Guglielmino, 2003; Watkins et al., 2004).

There are two different perspectives on e-learning readiness. One is about the organization and addresses the learning environment only. Borotis and Poulymenakou (2004, p. 1622) define it as “the mental or physical preparedness of an organization for some e-learning experience or action.” However, we take the second perspective and concentrate on the e-learning readiness of the individual learners only as we are interested in a human-centered approach. “Readiness” in that term means the competencies, aptitudes, and preferences of an individual to participate successfully in e-learning environments (Gay, 2018, p. 69). The proposition reflected in that definition that e-learning readiness of individuals is a multidimensional psychological construct is widely accepted. However, it is still under debate which dimensions are important (e.g., Demir and Yurdugül, 2015).

Different attempts to conceptualize e-learning readiness have been already made. In the following, we will give a brief overview.

First attempts to describe the readiness-concept of e-learning can be traced back in the 1990s. Some authors use e-learning readiness and online-learning readiness equivalent. Warner et al. (1998) for example, postulated a three-facet structure of online learning readiness: students’ preferences, digital confidence, and abilities for learning autonomously, which can be linked to the concept of self-directed learning (SDL; Guglielmino, 1977). Based on McVay (2000), Smith et al. (2003) focused on student behavior and attitudes. They suggested a two-factor structure, consisting of comfort with e-learning and self-management of learning. This attempt has been widely accepted and further developed by Bernard et al. (2004) with the aim to predict learning outcome, what was only partially successful. They postulated the following readiness-dimensions: beliefs about distance education, confidence in prerequisite skills, self-direction and initiative, and desire for interaction. Hung et al. (2010) proposed a five-dimensional model consisting of computer/internet self-efficacy, SDL, learner control, motivation for learning, and online communication self-efficacy. This is one of the most recent conceptualizations and considers motivational, regulatory, and personality-related aspects. Cigdem and Öztürk (2016) took up this concept bringing it into relationship with learners’ achievement. However, they found that students’ self-direction toward online learning was the strongest predictor for learning achievement, while motivation and computer/internet self-efficacy had surprisingly no significant effect.

In summary, the number of proposed e-learning readiness dimensions strongly varies and different dimensions have been identified as crucial depending on research group and underlying theories. However, some dimensions like self-regulatory skills are mentioned consensually. Findings about motivation and digital literacy are inconsistent with regard to their impact on outcome measures (e.g., Cigdem and Öztürk, 2016).

It has to be determined which variables constitute different dimensions of the concept of e-learning readiness of adult learners in continuing education. Furthermore, we argue that each dimension should be clearly defined taking recent educational research theories into account. Sticking to the definition of e-learning readiness from Gay (2018, p. 69) we are interested in learner characteristics in terms of competencies, aptitudes, and preferences in and on e-learning to reach that aim.

In the following part, we will give an overview about promising e-learning readiness dimensions derived from previous research findings and link them to educational learning theories to build up on a theoretical framework.

### Learner characteristics

#### Motivation

Motivation is known to be needed in all kinds of learning activities and accounts for one of the key success factors also in adult learning (e.g., Cho and Heron, 2015; Rothes et al., 2017; Mavropoulos et al., 2021). To start and maintain a learning process requires a certain level of motivation. A lack of motivation on the other hand can result in learning drop-out. An explanatory model for motivation in learning is based on the expectancy-value theory by Vroom (1964). According to that theory, expectancy means an individuals’ expectation for success. It has been adapted several times (e.g., Eccles, 1983, 1987; Pekrun, 2000). Expectancy beliefs can be positive or negative based on the learners’ perception about their own abilities and skills to master a learning task. By gaining experience in e-learning, expectancy beliefs can be modulated according to Wang et al. (2013). Value describes the perceived usefulness or importance of a learning task. Both components are known to contribute to learning success when positive. Doménech-Betoret et al. (2017) could prove, for example, that expectancy-value beliefs mediate the role between self-efficacy and satisfaction. Also, Artino (2008) and Diep et al. (2017) found that satisfaction can be explained by positive task value. Recent research findings by Lee and Song (2022) indicate that positive task value is associated with student engagement and influences learning persistence in Massive Open Online Courses (MOOCs).

But also the cost component of motivation was brought into focus. Cost means negative perceived aspects resulting from doing a task. For example, Barron and Hulleman (2015) proved cost as negative predictor for interest and achievement outcome. That is in line with results from Jiang et al. (2018), who also found a relationship between motivational cost and learning outcome. Perez et al. (2014) found that cost predicts even drop-out intention, what is supported by data from Francis et al. (2019) specifically for adult learners. To better understand the effects of motivational cost, Flake et al. (2015) argue that cost can be divided into subdimensions. They suggest “task effort” as the amount of work which has to be put into a task, “outside effort” as the additional effort for other tasks at the same time, “loss of valued alternatives” means that other preferred tasks cannot be done simultaneously and “emotional cost” describes the psychological state when doing a task what can lead to perceived stress. Based on these assumptions, highly perceived value, low-cost perceptions, and positive expectancy beliefs at the same time should lead to higher e-learning readiness levels and by that to a more positive learning outcome, measurable as course satisfaction.

An additional explanatory model for motivation is the achievement goal framework (Elliot and McGregor, 2001). According to that model, learners can follow performance and mastery goals, each subdivided into approach (achieve positive consequences of an action) and avoidance (avoid negative consequences of an action or non-action) tendencies. While performance goals are related to being better than others or perform exceptionally, mastery goals focus more on the learning process itself. The reward is learning new things. Achievement goals are proven to have an impact on academic outcome, for example (Hulleman et al., 2010; Goetz and Hall, 2013). However, findings are inconsistent. Mastery goals were found to be positively related to enjoyment that leads, in turn, to higher satisfaction and negatively related to boredom (Pekrun et al., 2006; Daniels et al., 2009; King et al., 2012; Goetz et al., 2016; Lüftenegger et al., 2016). For performance goals, correlations are still under debate. King et al. (2012) found that performance goals were also related to enjoyment, but only for the approach tendency. Recently, Pulkka and Budlong (2022) could prove an association between goal-orientation, self-efficacy, and individual learning preferences. Further research has to be conducted. Due to its relationship to learning satisfaction it is worth to examine whether the achievement goal framework contributes to define e-learning readiness.

#### Learning strategies, motivational regulation, and attitudes toward e-learning

Furthermore, Matos et al. (2007) postulated that a mastery goal-orientation is related to an increased use of learning strategies. More precisely, mastery goal-orientation predicts a better use of SRL and supportive online behavior according to Yeh et al. (2019). Learning strategies themselves, specifically SRL strategies, are advantageous for online learning (Yukselturk and Bulut, 2007; Jansen et al., 2022). This is supported by Steinkamp (2018) who found that a lack of organization and the ability to prioritize leads to struggling in online learning. Kizilcec et al. (2017) found that individual differences regarding the ability to manage the learning process autonomously exist. Wang et al. (2013) postulated that learners with previous e-learning experience, for example, had stronger SRL skills and therefore better learning outcomes. A similar concept is self-directed learning (SDL; Robotham, 1995; Pachnowski and Jurczyk, 2000). Sorgenfrei and Smolnik (2016) could show by a literature analysis that learner control has a direct effect on learning achievement. All three concepts are often used interchangeably and focus on the learners’ ability to manage their learning process with regard to cognitive, motivational, and emotional aspects of learning (Pintrich et al., 1993; Panadero, 2017). Motivational regulation (*cf.* Schwinger et al., 2007) as one crucial component of SRL, should be considered extensively when assessing learning strategies.

Another specific aspect of regulatory skills is time management. Especially e-learning requires appropriate time management skills, as the learning process is mostly controlled by oneself and many participants in continuing vocational education learn besides their regular work and social obligations without a designated time window. Thereby, we assume that bad time management can lead to increased motivational cost, as a ‘loss of valued alternatives’ or ‘outside effort’ becomes more likely. According to Gaspard et al. (2015), the general perception of cost increases over time. It is also known, that time management skills differ in (not only adult) e-learners (Alvarez-Sainz et al., 2019; Trentepohl et al., 2022).

But not only skills and abilities are related to course satisfaction as an outcome measure, but also the perception or preference for autonomous learning may be important. Zammit (2021) asked adult learners about their general perception of e-learning and could show that the higher level of autonomy was mostly experienced as advantageous, while at the same time distractions at home were negatively associated.

#### Personality

Besides motivation, learning strategies, and attitudes toward e-learning, it is widely accepted that personality traits can modulate learning outcomes of learners (Dabbagh, 2007; Del Valle and Duffy, 2007; Jensen, 2015). As research about personality traits in e-learning environments is scarce, the following concepts are mainly based on findings about learning in non-e-learning contexts.

For example, conscientiousness was identified as critical in predicting learning outcome (Trautwein et al., 2009; Conrad and Patry, 2012; Britwum et al., 2022). Mammadov (2022) conducted a meta-analysis finding relationships between openness to experience, extraversion, and agreeableness with learning achievement. While no significant correlations between neuroticism and extraversion with learning achievement were found by John et al. (2020). De Feyter et al. (2012) found positive effects for neuroticism on learning achievement when self-efficacy is high. Self-efficacy beliefs and attitude toward learning in general, have been identified for also having an impact on perceived satisfaction of the learners (Navarro et al., 2021). Ghorbani and Montazer (2015) even suggested an “automatic learners’ personality identifying system (ALPIS)” by linking online behavior, such as participating in chatrooms, to personality traits.

Additionally to the Big Five concept of personality, also perfectionism and self-efficacy in a broader sense of personality are known to have an impact on learning (Kurtovic et al., 2019; Güngör, 2020; Shaked and Altarac, 2022). Perfectionism can be described as a “multidimensional personality trait with adaptive and maladaptive qualities” (Hill et al., 1997, p. 3). The adaptive aspect can make a person strive for excellent outcome, also in learning. The maladaptive aspect can do exactly the opposite and lead to procrastination, for example (Capan, 2010). Kurtovic et al. (2019) found that academic achievement, self-efficacy, and adaptive perfectionism were negatively correlated with procrastination and maladaptive perfectionism was positively correlated with procrastination. So, this trait complex should also be considered.

Based on these findings, it is questionable if all of these facets linked to personality are important or if only some of them can be grouped to a global dimension of e-learning readiness.

#### Digital literacy

Digital literacy combines a set of skills, abilities, and attitudes. The European Framework for Digital Literacy (EFDL) defines it as “the awareness, attitude and ability of individuals to appropriately use digital tools and facilities to identify, access, manage, integrate, evaluate, analyze and synthesize digital resources, construct new knowledge, create media expressions, and communicate with others, in the context of specific life situations, in order to enable constructive social action; and to reflect upon this process.” (Martin, 2006, p. 155). This definition shows that digital literacy does not only mean the ability to work with technology, but also the attitude toward technology use and skills to integrate technology and social digital interaction which differ from face-to-face learning environments. This has an impact on learning outcome and has been researched extensively: Webster and Hackley (1997) found that learner’s attitudes toward the use of technology affect the success of e-learning. This was in the very beginning of the use of e-learning. Today most educational institutions use e-learning to enhance learning experience. It can be assumed that digital literacy has been increased over time by getting more familiar with technology use in general. But being confident with technology use does not necessarily mean that learners do not need support with digital learning strategies (Gurung and Rutledge, 2014). All these factors may have an impact on learning outcome and may account for one or more dimensions of e-learning readiness (e.g., Audrin and Audrin, 2022).

#### The present study

It is our aim to define e-learning readiness as comprehensive as possible. By adopting factors for successful learning from non-e-learning and e-learning settings and the review of previous attempts to define e-learning readiness, we aim to integrate these findings to a more holistic readiness approach. Previous research gave valuable insights into relevant aspects of e-learning readiness. However, we found the proposed models insufficiently empirically tested. According to McVay (2000) and Hung et al. (2010), especially aspects of predictive and convergent validity should be considered to also assess practical implications of the proposed instruments. Furthermore, they often lack a rigorous theoretical framework.

We also argue that due to digitalization, some aspects like having access to the internet may not be valid anymore and have to be adapted to current circumstances. Furthermore, some models have been tested on college students from one thematic field, only. Smith and Sadler-Smith (2003), for example, found the reliability for the specific learner group in vocational education for their model unsatisfactory.

We aim to contribute to closing these research gaps by developing a model for e-learning readiness, which is (a) based on a proper theoretical framework and (b) suitable for adult learners in continuing vocational education. Furthermore, we are interested in examining the dimensions derived from this with regard to their associations with learning satisfaction as a criterion for learning success and thereby delivering as an argument for the usefulness of the proposed dimensions in terms of to assess criterion-related validity.

## Materials and methods

To unveil dimensions of e-learning readiness in adult learners we conducted a study design combining qualitative and quantitative components. That design consisted of both, a semi-structured interview and, additionally, an online survey. By conducting the interview, we aimed to identify perceived challenges and benefits directly from the learners that provide information about motivation, regulation, and individual characteristics. The survey assessed biographical data, as well as a variety of personality traits, learning behavior, and motivational aspects to support at best the given answers quantitatively. We had no fixed preliminary hypotheses. Hence, the character of our study is clearly exploratory.

Using qualitative methods is often in line with smaller numbers of participants. We chose a specific sample that was about to complete a hybrid continuing vocational education training in the field of project management skills. Therefore, we followed the rules of purposeful sampling (Patton, 2002, p. 230). As we worked together in a research project, the recruitment process of participants was carried out by the Academy of the Ruhr-University. They focused on occupational social media networks and their own homepage to address participants. We neither had criteria for including nor for excluding participants. All participants gave informed consent prior participating in our research and were compensated with 20€ after completing both parts of the study.

### Semi-structured qualitative interview

With a total of *N* = 14 participants we conducted interviews. Due to COVID-19 restrictions, all interviews were carried out via online meeting platform Zoom. The participants were able to choose a preferred time window for that. All interviews were carried out by the same interviewer who was employed in the research project and holds a masters’ degree in psychology. For confidential reasons the interviewer was the only involved researcher in direct contact with the participants. This was a mandatory requirement, since we guaranteed the participants absolute anonymity in order to not risk their participation in the other parts of the study and in the course itself due to the rather small sample size. The interviews lasted on average 53 min.

We used a semi-standardized interview protocol which was based on a literature review and our theoretical implications regarding the aim of the study. By asking about challenges and benefits in e-learning we thought to gain insights about readiness-dimensions, as we assume that high levels of e-learning readiness are associated with the perception of fewer challenges and more benefits. Each interview consisted of five main parts.

1. After an introduction, we asked the participants about their intention to participate in a hybrid continuing education course. Main aspects were reasons for continuing education in general, reasons for a hybrid course format specifically, and the intended goals for taking that specific course.
2. Part two was about e-learning in general. Participants were encouraged to tell freely about a typical day when learning online. Aspects of interest were time spent with learning and differences between a learning day and a regular work day. After that, they were asked, what they consider attractive about online learning. An importance rating of all aspects has been carried out by the participants in case they mentioned more than one. This was followed by asking about challenges that the participants faced while learning online. Another importance rating took place like described above.
3. Part three of the interview was about the combination of e-learning and classroom setting of the hybrid course format. Attractiveness and challenges should be stated and rated by importance.
4. Next interview part was about motivational regulation. Situations were described by the interviewer using different vignettes. After reading out the vignette, the level of identification with the described situation was stated on a scale ranging from 1 (*very bad*) to 10 (*very good*). There were different aspects of interest: first, we asked about how the participants motivate themselves to start or continue with learning (motivational regulation) in case they have the expectation that a learning topic is hard versus boring to learn. Thereafter we asked specifically about perceived motivational cost before and during learning by having the participants describe their motivational regulation strategies and what they consider helpful to increase motivation.
5. Last part of the interview was about time management by also using vignettes. Participants stated their level of identification like described above. Thereafter, they were encouraged to describe their skills regarding time management and what could be helpful to improve them.

The interview ended by asking the participants if any questions have arisen and by thanking them for their participation.

After completion of the interviews every raw data audio file has been transcribed into written files by four research assistants. To improve the quality, every file was reviewed by another person to correct minor writing errors, however, changes regarding content have not been made. For confidential reasons every audio and written file has been encoded.

Thereafter, the corresponding author carried out a qualitative content analysis using a systematic, rule-guided method described by Mayring (2000). The aim is to build up a category system to give structure to the participants’ statements. This kind of analysis consists of different steps and is iterative (Schilling, 2006). First step was to paraphrase all content to make sure using a standardized style of language. After that, every statement was summarized and reduced by resuming the main aspect. A research assistant supported this process. Each and every statement then was categorized by a system emerged from our theoretical considerations. Based on that category system, we encoded all interview data using the software MAXQDA (VERBI Software, 2021). To make results transparent and replicable we established a coding manual in which all expected and actual categories are described by examples. As the carried-out analysis method is explicitly open to changes during the process, we added a subcategory for motivational regulation strategies that were mentioned. We discussed our final results within the research team and found all relevant information sufficiently processed.

### Online survey

A total of *N* = 35 participants completed the online survey using a survey software tool. Fourteen of the participants were female, and 21 were male. The average age was *M* = 37.66 (*SD* = 8.25). 60% of them have completed A-levels, and 27 participants are holding a university or at least a college degree. Four of them had a Ph.D. They worked for different companies in different occupational fields and branches and did not know each other before the course.

The first part of the survey took place after the first of two self-study phases, but before conducting the interview. For confidential reasons, all participants chose a number code to identify and to match the data of the survey parts after completion. The second part was carried out after the interview, but before the final course exam. The first part of the online survey was about the conditions of the participants’ taken continuing education course (e.g., “How many hours weekly do you spend on average on learning for this course?”). After that, participants were asked about biographical data such as age, gender, occupation and educational background. Response format differed based on the question.

The second part consisted of several standardized and well-established inventories which were partially slightly adapted (e.g., name of the continuing education course was inserted instead of *study major*) for better matching with our sample. Inventories were about personality traits, learning preferences, satisfaction measures, and general attitudes toward learning.

To assess participants’ personality traits, we used the *Big Five Inventory (BFI-K)* scale by Rammstedt and John (2005, see also Kovaleva et al., 2013). It is based on the Big Five dimensions (openness to experience, conscientiousness, extraversion, agreeableness, and neuroticism) and participants are asked about their level of agreement to different statements using 21 items (e.g., “I’m interested in many things”) with a five-point Likert scale ranging from 1 (*very true*) to 5 (*not true at all*).

Achievement goal-orientation was measured by an adapted version of the *2 × 2 Achievement Goal Framework* by Elliot and McGregor (2001). It is divided into mastery approach, mastery avoidance, performance approach, and performance avoidance goal-orientation and consists of 12 items (e.g., “I want to learn as much as possible from this class’’). Response format is a seven-point Likert scale ranging from 1 (*not at all true of me*) to 7 (*very true of me*).

Tendencies of perfectionism were measured by using parts of an adapted version of the *Frost Multidimensional Perfectionism Scale* (FMPS; Frost et al., 1990) by Stoeber (1998). We used the subscales *personal standards* (PS; 6 items; e.g., “I set higher goals than most people”) and *concern over mistakes and doubts* (CMD; 9 items; e.g., “I hate being less than the best at things”). Response format is a five-point Likert scale ranging from 1 (*not true at all*) to 5 (*very true*).

To measure general work satisfaction, we used the *KAFA* inventory by Haarhaus (2015) with a total of 30 items. On a five-point Likert scale ranging from 1 (*not true at all*) to 5 (*very true*), satisfaction with different dimensions of work satisfaction is rated by the participants. Dimensions are tasks (e.g., “My work tasks are exciting.”), colleagues (e.g., “My colleagues are pleasant.”), development (e.g., “My development possibilities are good.”), salary (e.g., “My salary is satisfactory.”), supervisor (e.g., “My supervisor is fair.”), and an over-all rating. An item for over-all satisfaction is: “All over, my job is satisfying.”

Different aspects of stress have been measured by using the *Perceived Stress Questionnaire* (PSQ; Levenstein et al., 1993; Fliege et al., 2001). It consists of the following subscales: joy (e.g., “I am full of energy.”), worries (e.g., “I worry a lot.”), tension (e.g., “I have problems to relax.”) and requirements (e.g., “I have the feeling that too many demands are being made on me.”) and a total of 20 items. Response format is a four-point Likert scale ranging from 1 (*hardly ever*) to 4 (*usually*).

Thereafter, self-efficacy has been measured by an adaptation of a German inventory by Schwarzer and Jerusalem (1999, 2003) with a total of 10 items. Response format is a four-point Likert scale ranging from 1(*not true*) to 4 (*exactly true*). Exemplary item: “I can find a solution for nearly every problem.”

Furthermore, two facets of test-anxiety have been assessed by an adaption of the German *Test-anxiety Inventory* (TAI-G; Hodapp, 1991) by Schwarzer and Jerusalem (1999; see also Musch and Bröder, 1999). Both components of test-anxiety are measured by 5 items each like “I am excited.” (excitement component, TAI-E), “I am concerned that something might go wrong.” (worries component, TAI-W). Response format is a four-point Likert scale ranging from 1(*not true*) to 4 (*exactly true*).

Learning strategies were measured by using the *LIST* inventory (Wild and Schiefele, 1994) by Boerner et al. (2005) consisting of a total of 88 items. We used the subscales effort (e.g., “I do not give up, even when the subject is difficult or complex.”), concentration (e.g., “When I’m learning, I’m easily distracted.”), time management (e.g., “When I learn, I stick to a specific schedule”), learning environment (e.g., “My workplace is designed in such a way that I can find everything easily.”), literature use (e.g., “I collect missing information from different sources, e.g., notes, books, journals.”), colleagues (e.g., “If something is not clear, I ask a fellow colleague for advice.”), organization (e.g., “I summarize the most important content as a reminder.”) and goal-setting and planning (e.g., “Before I start learning, I think about how I want to learn.”). Response format is a six-point Likert scale ranging from 1 (*not true at all*) to 6 (*very true*).

For measuring drop-out intention we used an adapted version of an inventory by Dresel and Grassinger (2013). It consists of five items (e.g., “I often think about dropping out of my current course”) and response format is a six-point Likert scale ranging from 1 (*absolutely false*) to 6 (*absolutely true*).

To measure procrastination we used an adapted version of the *Tuckman Procrastination Scale* (TPS-D; Tuckman, 1991; Stoeber, 1995) Participants rate their level of agreement with the 16 items (e.g., “I unnecessarily procrastinate on completing Six Sigma Green Belt course tasks, even when it is important”) on a five-point Likert scale ranging from 1 (*not true at all*) to 5 (*very true*).

To assess the perceived task value in terms of course utility, we used 6 items from the *Motivated Strategies for Learning Questionnaire* (MSLQ) inventory by Pintrich et al. (1991; e.g., “I think I will be able to use what I learn in this course in other courses”). Response format is a seven-point Likert scale ranging from 1 (*not at all true of me*) to 7 (*very true of me*; see also Credé and Phillips, 2011).

Final aspect of interest was course satisfaction which was measured by a modified version of the *German Satisfaction Inventory* by Westermann et al. (1996, 2018). Participants are asked how much they agree with the statements ranging from 1 (*not true at all*) to 10 (*absolutely true*). Three facets, each measured by 3 items, are differentiated: satisfaction with learning content (SLC; e.g., “Overall, I’m satisfied with the Six Sigma Green Belt Hybrid training”), satisfaction with course conditions (SCC; e.g., “There is too little attention paid to the concerns of the participants at the Academy of the Ruhr University”) and satisfaction with coping with course burdens (SDCB; e.g., “I have difficulties balancing Six Sigma Green Belt Hybrid continuing education with other commitments”).

## Results

### Interview data analysis

The interview data revealed different themes of perceived challenges and benefits and how participants cope with them. We divided these themes according to our theoretical considerations into the main categories: motivation, learning strategies (motivational regulation), personality, and digital literacy (especially the ability to apply coping strategies related to the need for interaction and communication with others). Different from the theory part, time management and learning environment have emerged as new separate categories.

### Motivation

Challenges and benefits related to motivation were most mentioned. On the one hand, there was a total of 220 statements about the highly perceived value of the e-learning format of the course. These were divided into sub-categories based on the Expectancy-Value framework by Flake et al. (2015). Most often utility value was mentioned (98 times in total), followed by the intrinsic value (96 times in total). Only seven statements were about attainment value and a total of 19 statements could not be clearly assigned to one sub-category only.

A question revealing hints about motivation was:

“What do you consider attractive about online learning?”

For example, Participant 1 answered: “In any case, flexible scheduling. So from the time when you look at which topic, that you can spread it out over the day.” (utility value), while participant 2 referred to intrinsic value by saying: “Depending on how the modules are structured, you can also take into account your own learning preferences.” A total of 12 participants addressed positive expectancy beliefs. They mentioned former experience with e-learning as motivating. Participant 3 answered: “The first has the biggest one, also because I think I’m quite ok with learning and I trust that I can manage it in time.”

Cost-related statements were mentioned 171 times in total. Thereof were 42 about loss of valued alternatives, 31 about effort, 44 about outside effort, and 53 about affective cost.

A question that could be linked to motivational cost was, for example:

“To what extent do you face challenges when learning online?”

Also, cues for mastery and performance goal-orientation according to Elliot and McGregor (2001) have been found. Participant 4 told us: “When I come (to the course) and am not prepared everybody is laughing at me.” “That statement refers to performance-avoidance self-talk. While “I just want to reach a certain goal and when I have started and already have made good progress,” was identified as performance-approach self-talk. Only two mastery-approach self-talk cues could be identified in the interview data.

### Learning strategies (motivational regulation)

We asked participants about how they motivate themselves for learning. Thereby, we gained insights about their regulatory skills. Specifically, we asked participants to imagine the following situation:

“Please imagine you are in the process of learning online and cannot motivate yourself to start/continue learning, because you find the learning material very difficult (condition 1) or boring (condition 2). Which options do you know to motivate yourself to start/continue learning?”

From all mentioned motivational regulation strategies most statements were about setting learning goals, learning environmental control, and help-seeking behavior. No differences regarding boredom or difficulty condition could be identified. An overview about the mentioned motivational strategies can be found in Table 1.

**Table 1:** Motivational regulation strategies.

Motivational regulation strategy	*N* participants	Total count of statements
Proximal goal-setting	11	76
Environmental control	12	51
Prioritizing	12	49
Help-seeking behavior	11	47
Enhancement of personal significance	9	25
Enhancement of situational interest	8	17
Performance-avoidance self-talk	6	11
Performance-approach self-talk	3	10
Self-consequating	4	10
Mastery-approach self-talk	1	2

*N* = 14.

### Personality

As we did not conceptualize questions specifically about personality traits, we screened the interview data for self-descriptive statements that allow conclusions about the participants’ personality. Statements about conscientiousness and extraversion could be found. Most statements were about conscientiousness. The answers were not linked to a specific question, but could be traced back to the boredom condition mentioned in the above section. Participant 11 answered: “You have to overcome your inner weakness,” for example. Cues for extraversion were mainly related to help-seeking behavior or the need for interaction with peers. For example, one participant said about the hybrid learning context: “…that you are still not quite so isolated and still get to know other people.” Another one told us: “Mhm, the reasons are probably obvious. I really like working with people, I also really like getting to know other people and their perspectives, and especially for me in this context, I, of course, also got to know people there with whom I rarely have anything to do in normal everyday work…”

### Time management

As cues for time management skills were very salient, we chose to give them an extra category. With a total of 61 statements at least 9 participants mentioned time management as a challenge in e-learning. They admitted that they have the tendency to procrastinate.

“Then I’ll do it on another day, when there is more time.”

“I would put off the whole thing until my time management says ‘it’s actually late now, you have to start now.”

“I have started now, because in 2 weeks there is the next attendance meeting.”

### Digital literacy

In the interview data, we did not found any cues that confirm or refute our theoretical assumptions about skills in technology use being mandatory for high levels of e-learning readiness. Not a single participant reported about it, when asked about challenges that occurred during learning.

However, at least 11 participants mentioned the lack of interaction while e-learning as challenging. More precisely, they criticized that there were too few options for professional exchange with peers. Furthermore, they claimed that it would be helpful to have a teacher available for feedback and if questions arise. We conclude that coping strategies in dealing with the need for interaction and communication can be important to describe a high degree of e-learning readiness.

One participant said: “That was missing, the other participants were also waiting for a platform - somewhere where you can ask questions in the chat or whatsoever.” Another one told us: “I like doing my thing on my own and learning on my own, but I still found it very exciting to get to know the others in the face-to-face event, from which areas they come, how they can apply what they have learned, how it works for them runs to exchange,” for example.

### Learning environment

At least 13 participants mentioned more or less successful dealing with their learning environment. For example, Participant 3 told us that learning at home is easier:

“But it did not work as well as at home, because at work someone keeps coming to the desk and someone always has some issues.” In contrast, Participant 9 says: “So I make sure that the child sleeps and leaves me alone and I tell my husband to leave me alone.”

### Survey data analysis

All data analysis was conducted using IBM SPSS (Version 28.0). Due to the rather small sample size and since some of the variables were not normally distributed, we conducted non-parametric tests, where applicable. According to Kim and Choi (2021), we set the significance level at *p* ≤ 0.10 suitable for exploratory research.

### Descriptive analyses

Table 2 contains descriptive statistics and scale reliabilities (Cronbach’s alpha) for all variables assessed in the survey. Most instruments showed satisfactory Cronbach’s alpha reliabilities. Only BFI-K agreeableness and satisfaction with course conditions showed reliabilities below *α* = 0.60.

**Table 2:** Means, standard deviations, range and scale reliabilities (Cronbach’s α).

	Mean	Standard deviation	Range	Cronbach’s alpha
BFI-K agreeableness	3.22	0.65	3.00	0.52
BFI-K conscientiousness	4.09	0.50	1.75	0.65
BFI-K extraversion	3.61	0.71	2.75	0.79
BFI-K neuroticism	2.65	0.78	3.50	0.78
BFI-K openness to experience	3.82	0.70	2.60	0.72
LIST effort	4.41	0.64	2.63	0.70
LIST concentration	4.15	0.98	3.50	0.91
LIST time management	3.27	1.22	4.50	0.84
LIST Learning environment	4.52	0.78	3.33	0.69
LIST goal-setting/planning	4.05	0.75	3.33	0.60
LIST literature	4.24	1.25	4.50	0.76
LIST organization	3.60	1.14	4.67	0.87
LIST colleagues	2.69	1.24	3.75	0.80
Mastery goal-orientation approach	6.32	0.76	2.33	0.71
Mastery goal-orientation avoidance	5.17	1.48	6.00	0.78
Performance goal-orientation approach	3.90	1.83	6.00	0.91
Performance goal-orientation avoidance	4.09	2.02	6.00	0.91
Satisfaction with work tasks	3.75	0.67	2.40	0.78
Satisfaction with colleagues	4.08	0.70	2.80	0.89
Satisfaction with development	3.51	1.14	4.00	0.94
Satisfaction with payment	4.07	0.85	3.00	0.90
Satisfaction with supervisor	3.96	0.85	3.80	0.91
Over-all satisfaction with job	4.07	0.71	2.80	0.90
Self-efficacy	3.00	0.43	2.10	0.90
Test-anxiety excitement	1.73	0.60	2.00	0.99
Test-anxiety worries	2.02	0.77	2.80	0.86
Stress total	2.44	0.51	2.05	0.92
Stress joy	2.39	0.61	2.40	0.80
Stress tension	2.58	0.65	2.60	0.83
Stress worries	1.93	0.64	2.60	0.84
Stress requirements	2.87	0.56	2.00	0.75
Perfectionism PS	3.26	0.77	3.57	0.81
Perfectionism CMD	2.07	0.71	3.08	0.90
Task value	6.19	0.81	3.17	0.88
TPS-D	2.17	0.63	2.38	0.89
Drop-out intention	1.77	0.92	4.00	0.75
Satisfaction with course content	8.19	1.60	8.33	0.77
Satisfaction with course conditions	7.93	1.69	6.00	0.50
Satisfaction with coping with course burdens	6.98	2.00	7.67	0.79

*N* = 35.

By calculating means and standard deviations we got a first impression about how the test values are distributed and thereby, if they are potentially suitable for indicating e-learning readiness.

As a next step, we conducted a correlational analysis by calculating correlations between indicators of potential e-learning readiness dimensions and the three facets of course satisfaction as relevant criteria for learning success (see Table 3). By that, we aimed to support the findings from the interview data and check practical implications of the assumed dimensions.

**Table 3:** Correlations between indicators of potential e-learning readiness dimensions (inventories) and course satisfaction as a criterion for learning success.

Inventory	Satisfaction with coping with course burdens	Satisfaction with course conditions	Satisfaction with course content
BFI-K agreeableness	0.292^*^	0.315^*^	0.170
BFI-K conscientiousness	0.047	0.182	0.356^**^
BFI-K extraversion	0.163	−0.211	−0.020
BFI-K neuroticism	−0.190	0.014	−0.110
BFI-K openness to experience	0.322^*^	0.169	0.215
LIST effort	0.180	0.214	0.215
LIST concentration	−0.003	0.213	−0.003
LIST time management	−0.014	0.142	0.139
LIST learning environment	0.072	0.188	−0.292^*^
LIST goal-setting/planning	−0.015	0.049	−0.038
LIST literature	0.132	0.351^**^	0.016
LIST organization	0.147	0.227	0.151
LIST colleagues	0.229	−0.218	−0.179
Mastery goal-orientation approach	0.234	0.306^*^	0.015
Mastery goal-orientation avoidance	0.314^*^	0.240	0.054
Performance goal-orientation approach	0.028	−0.186	0.092
Performance goal-orientation avoidance	0.163	0.092	0.152
Self-efficacy	0.320	−0.043	0.213
Test-anxiety excitement	−0.413^**^	−0.096	0.052
Test-anxiety worries	−0.361^**^	−0.144	0.163
Stress total	−0.406^**^	0.118	−0.052
Stress joy	−0.268	0.123	−0.580
Stress tension	−0.409^**^	0.075	0.004
Stress worries	−0.254	0.057	0.119
Stress requirements	−0.394^**^	0.072	0.081
Perfectionism PS	−0.227	−0.092	0.106
Perfectionism CMD	−0.371^**^	0.004	0.021
Task value	0.411^**^	0.215	0.475^***^
TPS-D	−0.225	−0.370^**^	−0.179
Drop-out intention	−0.593^***^	−0.183	−0.348^**^
LIST item: I try to organize the material in such a way that I can memorize it well.	0.076	0.407^**^	0.009
LIST item: I have the most important documents to hand.	0.348^**^	0.165	−0.096
LIST item: I orientate myself on the working instructions of the teaching materials.	0.334^**^	0.471^***^	0.177

*N* = 35. ^*^p < 0.10; ^**^p < 0.05; ^***^p < 0.01.

For satisfaction with coping with course burdens positive correlations have been found with mastery goal-orientation, self-efficacy, task value, agreeableness and openness to experience. Negatively associated with satisfaction with coping with course burdens were perfectionism (CMD), stress, test anxiety, and drop-out intention.

For satisfaction with course conditions, positive correlations have been found with mastery goal-orientation, Literature, and Agreeableness. Negative correlations between satisfaction with course conditions have been only found for procrastination.

For satisfaction with course content, positive correlations could only be found with task value and conscientiousness. Satisfaction with course content was negatively correlated with LIST learning environment and drop-out intention (see Table 3).

## Discussion

In the following sections, we will be discussing the findings from both, the interview and the survey data in the light of previous research findings. After that, we will focus on the limitations of the study and give an outlook on future research directions and the current state regarding planning an onward study.

Overall, we have reached our research aim. Based on genuine perceptions of participants of a continuing vocational education course and supported by the survey data we were able to identify several possible dimensions of e-learning readiness. We could even confirm their practical implication by linking them to learning success in terms of course satisfaction.

### Key indicators of e-learning readiness

#### Motivation

Our main exploratory field was the interview data. By asking the participants directly for perceived challenges and benefits during their e-learning process we got a more genuine idea of dimensions that are contributing to describe e-learning readiness.

The most mentioned category was motivation. All participants were able to easily reflect on perceived benefits related to the value component of motivation. They did not only refer to the course content, which they have chosen voluntarily, but also to the hybrid course format as a crucial benefit for their learning process. This can be explained by the greater perceived choice regarding the flexibility of learning. Participants were mostly free when and where to learn. We assume that the higher degree of autonomy leads to a better balance between learning and other obligations. This in turn, should contribute to course satisfaction, what is supported by findings from Castro and Tumibay (2021), for example. They found by conducting a meta-analysis that flexibility regarding learning schedules is perceived beneficial, especially by adult learners with multiple obligations and high work demands besides learning. We conclude, that highly perceived course value, especially regarding the digital or hybrid course format, indicates high levels of e-learning readiness. However, Quesada-Parallès et al. (2019) did not find significant differences in perceived task value when comparing online and attendance learning settings, what supports our assumptions that the course format is more relevant in terms of motivation.

Also, motivational cost was mentioned often. Statements on affective cost were mentioned most frequently. Besser et al. (2022) draw similar conclusions: they found high levels of stress and negative mood in online learners. Outside effort and the loss of valued alternatives were similarly often mentioned, while effort had the fewest statements. This clearly shows that not the learning process itself is perceived stressful, but the conditions besides learning are. We assume that high levels of perceived motivational cost contribute to low e-learning readiness and that strategies coping with motivational cost contribute to e-learning readiness.

As nearly all participants addressed positive expectancy beliefs as motivating, it can be assumed that previous e-learning experience is beneficial for the learning process and contributes positively to e-learning readiness. However, Berweger et al. (2022) could recently show a moderating effect of perceived cost on the relationship between expectancy beliefs and frustration in online learning. Therefore, the expectancy-value framework should be carefully examined regarding other moderating effects in future research.

Regarding goal-orientation of the learners, our findings are inconsistent. Performance goal-orientation was more salient in the interview data than mastery goal-orientation. However, we did not differentiate between approach and avoidance tendencies. The additional findings from the survey support this only to some extent. Mastery-approach goal-orientation had the highest mean, followed by mastery-avoidance goal-orientation. By using a correlational design, we could interlink a proposed dimension to the criterion of course satisfaction which further underlines its importance. Our data shows that a mastery goal-orientation is linked to higher course satisfaction regarding course conditions and coping with course burdens. Contrary to the interview data, performance goal-orientation had lower means and showed no effect on satisfaction. This can be explained by a lack of self-reflection skills of the participants or a lack of the ability to clearly discriminate both concepts, as they sound very similar in the survey. As there is no extensive interaction between the learners, less social comparison is carried out and performance goals may be less triggered. Additionally, the interview was carried out before an attendance-meeting (trigger for performance goal-orientation), while the survey took place independent from any attendance meetings. Recent research findings regarding goal orientation also indicate no consistency. Zhou and Wang (2019) found indirect relationships between performance-approach and mastery goal-orientation and academic performance. However, Güler (2017) found only low effect sizes for goal-orientation on learning outcome.

Summing up our findings about motivation, they are in line with the proposed theoretical framework described in the introduction part. High levels of perceived value, low-cost perceptions, and positive expectancy beliefs as well as a mastery goal-orientation have been identified to contribute positively to e-learning readiness. It needs further research how to integrate these findings to one comprehensive dimension of e-learning readiness.

#### Personality

Within the interview data, we found some cues for the personality trait conscientiousness. Participants mentioned it with regard to focus on the learning goal and perseverance, even when problems occur.

In the survey, we have broadened the topic and assessed besides the Big Five personality traits also perfectionism, self-efficacy, and procrastination behavior as facets linked to personality. For perfectionism, we found that lower levels of concern over mistakes and doubts were linked to higher satisfaction with coping with course burdens, but not course conditions. Madigan (2019) also argued in his meta-analysis that maladaptive aspects of perfectionism are linked to numerous negative outcomes such as depression, burn-out, or procrastination. According to that, low levels of procrastination were associated with higher satisfaction with course conditions in the present study.

Both perfectionism and procrastination contribute to learning outcome. We found also self-efficacy positively related to satisfaction with coping with course burdens. This is in line with findings by Brown et al. (2011), who argued that individuals with higher scores of conscientiousness had higher self-efficacy beliefs. Navarro et al. (2021) also confirmed the impact of self-efficacy beliefs on course satisfaction.

Regarding Big Five personality traits we found that agreeableness is positively linked to satisfaction with both, course conditions and coping with course burdens. Openness to experience was positively related to satisfaction with coping with course burdens, only. While openness to experience can be understood as positive attitude toward new experiences also in learning, it can be assumed that also coping strategies are used more creatively and therefore lead to higher satisfaction. Agreeableness, however, was not associated with satisfaction in the present study, but is known to have positive effects on work satisfaction, for example (Organ and Lingl, 1995; Judge et al., 1999). This may be the same mechanism for course satisfaction. Support for this hypothesis was described by Patitsa et al. (2021) who found that higher levels of overall satisfaction with online learning were linked to higher levels in all Big Five personality traits with neuroticism exempted.

It has to be examined how these findings can be summarized to a readiness-dimension. Especially perfectionism should be taken into account by explaining how it might be different to conscientiousness. Also, other personality traits should be assessed by using additionally job-related inventories.

However, our data support the theoretical assumptions about personality traits contributing to e-learning readiness.

#### Learning strategies

Learning strategies in the interview part were coded as SRL-cues. We focused on motivational regulation, specifically. Goal-setting, environmental control abilities, and help-seeking behavior were most mentioned. So, the organization of learning seems to be indeed relevant for the participants.

Findings from the survey also indicate that organizational strategies contribute to course satisfaction. It could be shown that the LIST subscale literature is related to higher satisfaction with course conditions. The learners’ ability to do research on their own in order to gain new information in case they do not understand a topic, is a crucial aspect in e-learning readiness (e.g., I collect missing information from different sources, e.g., notes, books, journals). However, it was surprising that no significant correlations could be found for the LIST subscale organization itself. An additional correlational analysis on item level of the subscales organization and learning environment revealed correlations for two items (“I follow the work instructions in the teaching material” and “I arrange the material in such a way that I can memorize it easily”) with satisfaction with course conditions and for one item out of the subscale learning environment (“I have the most important documents ready at the workplace”) with satisfaction with coping with course burdens. Partially, the present findings are in line with previous research (e.g., Bernard et al., 2004; Hung et al., 2010).

We assume that using the LIST inventory is not suitable for conceptualizing organizational strategies as a readiness-dimension. Therefore, we suggest to generate new items in future research.

In summary, managing the learning process autonomously (with regard to learning strategies and motivational regulation) determines higher levels of e-learning readiness according to our theoretical considerations. This is supported by our data.

#### Time management

Time management was very salient in the interview data. About 30 statements about procrastination were identified. It is questionable, if time management accounts for a separate dimension or if it is related to organizational strategies or personality. Survey data did not support either one or another direction. The LIST subscale time management was on average level and showed no connection to satisfaction measures. Also, surprisingly the TPS-D scale did not reflect the mentioned problems regarding time management. The procrastination level of the participants was below average. Possibly, participants were not able to link proper time management with procrastinating behavior or they had the knowledge about time management strategies, but were not able to apply them correctly. However, it has been postulated by Wladis and Samuels (2016) and Martin et al. (2020) that time management can account for a separate dimension, which has to be tested.

#### Digital literacy

Even though we proposed a wide definition of digital literacy in the introduction part, it does not cover the aspects emerged from the interview data fully that we relate to digital literacy. In past research digital literacy was given a high priority with regard to appropriate technology use (see Martínez-Bravo et al., 2020 for a meta-analysis). We screened the interview data and could not find any cues related to learning challenges with technology use. However, we did find statements about the lack of interaction and struggling with communication within the learning group and with the teacher. This is in line with recent findings by Delcker and Ifenthaler (2022) who found the lack of feedback and options to interact in e-learning as challenging for learners. With the growing shift toward digitalization in learning and work environments, we assume that the original dimension of digital literacy is no longer valid, especially for adult learners. Instead of focusing on skills and abilities of technology use, we suggest that examining digital communicational skills and assertiveness is more useful in terms of e-learning readiness. Referring to the concept of literacy, it is necessary to further assess abilities and skills that contribute to coping with these needs. As we did not conceptualize that within the survey, it needs further research how to assess the proposed sets of abilities and skills. It can be assumed that different levels of coping abilities with need for interaction and the assertiveness in digital communication have an impact on learner’s readiness levels and also on course satisfaction.

#### Learning environment

The coded dimension “learning environment” from the interview data has been mentioned by 13 out of 14 participants. The quotations suggest that learning environment corresponds to the ability to create an undisturbable learning atmosphere by avoiding distractions effectively (see Schwinger et al., 2009). It has to be tested, if this accounts for a separate dimension or can be summarized to other regulation strategies.

#### Demographics

By also collecting biographical data, we gained information about the demographical background of the participants. We found a wide range of age among them in the sample. This fact by its own may influence their individual readiness levels as it is known that learning strategies could change over time (Akbar, 2020). Also, motivation regulation strategies are changeable based on learning subject, for example (Schwinger and Stiensmeier-Pelster, 2012). The learning environment of adult learners could be different as they may have another family background and social obligations (Romero and Barberà, 2011), which in turn may influence perceived motivational cost. While biographical data is not suitable for accounting for a distinct dimension of e-learning readiness, it can be useful to have additional information ready which is needed for standardization of new measures. Due to the small sample size, we were not able to verify differential results for different age groups.

### New research insights and practical implications

Some of the above-discussed dimensions are in line with previous research findings, others only emerged from the present data.

The most consistent dimension is motivation. Many previous attempts to conceptualize e-learning readiness took motivation into account (e.g., Hung et al., 2010; Cigdem and Öztürk, 2016). However, the data indicates that not all facets and frameworks of motivation contribute equally to e-learning readiness. Focusing on the relevant subdimensions of the motivation framework only is one of the key findings of this study. Personality traits were underrepresented in previous research. While it is widely accepted that personality traits have an impact on learning outcome (e.g., Mammadov, 2022), research about the relationship between those personality traits and e-learning readiness is lacking. Therefore, the present study contributes to shed light on the interlink between a learners’ personality and e-learning readiness by conducting a broader approach of the personality concept consisting of the Big Five dimensions, perfectionism, and procrastination. Learning strategies, conceptualized as abilities (Warner et al., 1998) or skills (Bernard et al., 2004) have been also taken up by Hung et al. (2010) and Cigdem and Öztürk (2016). However, different to them, we strongly emphasize to concentrate on motivational regulation strategies as crucial part of organizational learning management strategies, as these were very salient in the interview data. This is, to the best of our knowledge, unique of our research approach. Hence, it is necessary to completely redefine the dimension of learning strategies.

As discussed in the above section, we suggest time management to be a distinct dimension of e-learning readiness and agree with research findings by Wladis and Samuels (2016) and Martin et al. (2020). Based on the inconsistent findings from the present survey compared to the interview data, it needs further research on how to address the gap between learners’ theoretical assessment of their own time management skills and the rather poorly rated time management reported in the learning situation itself.

In previous research managing the learning environment has been summarized to learning strategies mostly (e.g., Smith et al., 2003; Hung et al., 2010). Another interesting and broader approach to learning environment comes from the field of environmental psychology. Ng (2021) conducted research on the physical learning environment of distance learners. Findings indicate that not only social aspects like interacting with people around the individual learning space, but also physical aspects like spatial requirements or ambient features have an impact on learning. This view on learning environment is more in line with our data. Therefore, we propose to detach learning environment from the dimension of learning strategies and consider it separately by broadening the topic.

With the ongoing shift toward digitalization and the necessity for learners to adapt to digital learning environments, our research approach paves the way for recommendations for education providers. By measuring e-learning readiness adult learners can be characterized and possible challenges are unveiled before taking a course. This gives the opportunity to adapt courses for the needs of their participants. Thereby, indicators of learning success such as course satisfaction and learning achievement can be promoted.

### Limitations and outlook on future onward research

As a next step, we need to operationalize the identified dimensions of e-learning readiness by constructing an appropriate item pool. Therefore, we will be evaluating our findings regarding the theoretical framework and test our model statistically afterward.

However, our research has some limitations. As our sample is from one specific continuing vocational education course only, it would be beneficial to take a wider range of occupations into account to generalize our results in future research. Even though the present study is of exploratory character and in line with recommendations for qualitative research, the sample size was quite small. Due to the confidential reasons resulting from that, we were not able to match the interview data with the survey data, as we have guaranteed anonymity to the participants.

It should also be taken into consideration whether and how the COVID-19 pandemic could have had an impact on perceived time management skills or the attitude toward learning, for example. We did not assess changes in working conditions of the participants due to the pandemic, e.g., cutting work times, changes in work load or exemptions from work.

As learning in continuing vocational education is work-related, it could be useful to add work-related inventories. For assessing personality traits, for example, we suggest the Business-focused inventory of personality by Hossiep and Krüger (2017). The LIST inventory has not provided satisfactory results for learning strategies. Taking the results of the interview data into account, it is necessary to redefine the category of learning strategies. Motivational regulation strategies need to be considered more in-depth, as well as time management and learning environment.

The present study underlines the need for a new inventory that is applicable to a wide range of occupational fields. Previous measures were lacking an appropriate theoretical framework or were inconsistent in defining the proposed dimensions. Furthermore, they did not focus on adult learners and it is questionable if they are applicable for this group at all. The needs of adult learners like having other social obligations, limited resources or deviating learning preferences should be taken into consideration. Currently, we are in the process of generating item pools for each extracted dimension of e-learning readiness in order to develop a new instrument to assess the proposed construct. The new instrument then will be piloted and validated among participants in different industrial and scientific occupational fields as a next step.

## Data availability statement

The raw data supporting the conclusions of this article will be made available by the authors, without undue reservation.

## Ethics statement

The studies involving human participants were reviewed and approved by the Ethics committee of the Faculty of Philosophy and Educational Research of the Ruhr-University Bochum (Approval number: EPE-2022-013). The patients/participants provided their written informed consent to participate in this study.

## Author contributions

AS contributed to collect and prepare the data. VL analyzed the data, conceptualized and wrote the draft, and was responsible for the project. JF and JW contributed to revise the draft and gave conceptual advice. KT and LF contributed to discuss the data. All authors have approved the submitted version of the draft.

## Funding

This research was supported by the German Federal Ministry of Education and Research (Bundesministerium für Bildung und Forschung, BMBF, grant number: 01JD1907B).

## Conflict of interest

The authors declare that the research was conducted in the absence of any commercial or financial relationships that could be construed as a potential conflict of interest.

## Publisher’s note

All claims expressed in this article are solely those of the authors and do not necessarily represent those of their affiliated organizations, or those of the publisher, the editors and the reviewers. Any product that may be evaluated in this article, or claim that may be made by its manufacturer, is not guaranteed or endorsed by the publisher.
